# Unresponsive Intractable Chronic Headache With Sitagliptin

**DOI:** 10.7759/cureus.2537

**Published:** 2018-04-25

**Authors:** Ahmed Zaghloul, Corina Iorgoveanu, Andrew Polio, Aakash Desai

**Affiliations:** 1 Internal Medicine, University of Connecticut Health Center, Farmington, USA

**Keywords:** sitagliptin, diabetes, headaches

## Abstract

Sitagliptin is an anti-diabetic medication within the dipeptidyl peptidase 4 (DPP4) inhibitor class used as a single agent or in combination therapy. It is a well-studied and well-tolerated medication with commonly reported adverse events of upper respiratory tract infections, nasopharyngitis, headache, and gastrointestinal (GI) upset. Post-marketing reports have also identified associations with acute pancreatitis and joint pain. Here, we report a case of type II diabetes with chronic headache which resolved rapidly after discontinuation of sitagliptin. Our case demonstrates the need for continuous monitoring and post-marketing surveillance for drugs with tolerable side effect profile. Also, increasing patient and physician awareness of long-term side effects of these commonly used medications is essential for adequate patient safety and quality care.

## Introduction

Sitagliptin is an anti-diabetic medication within the dipeptidyl peptidase 4 (DPP4) inhibitor class which gained approval in the United States in 2006 for use in type II diabetes either as mono- or combination therapy. It is a well-studied and well-tolerated medication with commonly reported adverse events of upper respiratory tract infections, nasopharyngitis, headache, and gastrointestinal (GI) upset [1]. Post-marketing reports have also identified associations with acute pancreatitis and joint pain. Here, we report a case of type II diabetes with chronic headache which resolved rapidly after discontinuation of sitagliptin.

## Case presentation

**Figure 1:**
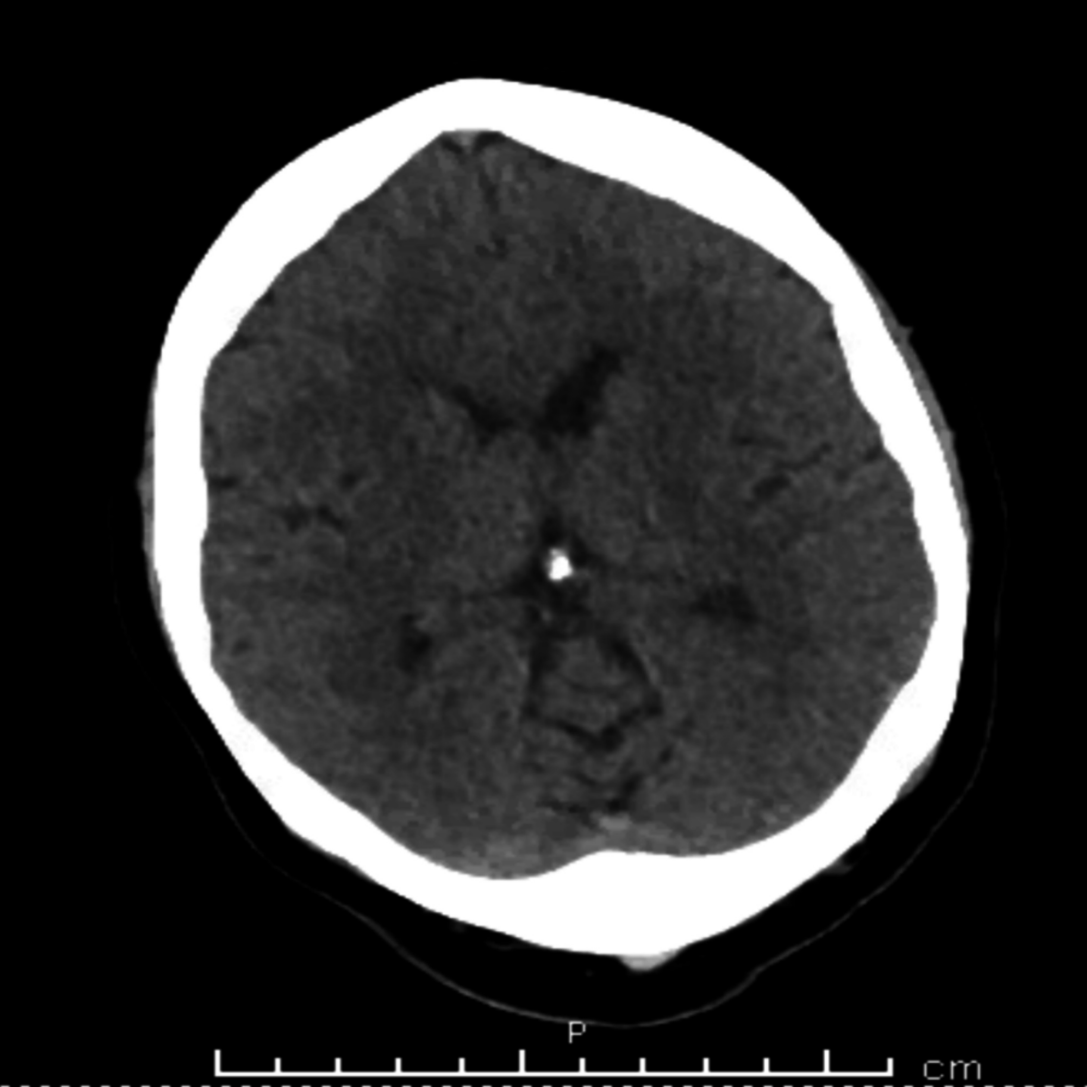
Computed Tomography (CT) Scan of the Head

A 51-year-old woman with a history of morbid obesity, obstructive sleep apnea on nocturnal continuous positive airway pressure ventilation, and non-insulin dependent diabetes mellitus presented to the urgent care clinic with an unresponsive intractable chronic headache for almost a year. The headache was 7/10, intermittent, non-radiating, throbbing, the frontal headache lasting for almost a year and was thought to be secondary to post-concussion syndrome given a history of head trauma one year ago with no loss of consciousness. At the time, computed tomography (CT) scan of the head was unrevealing (Figure 1). Over the past year, the patient had visited the emergency department multiple times with a severe headache. Secondary causes of headache such as severe hypertension, pharyngitis, sinusitis, meningitis as well as tumor, subdural hematoma, and hydrocephalus were ruled out. Laboratory work up was unrevealing with a normal ESR and CRP. Magnetic resonance imaging (MRI) scan and lumbar puncture were both negative. In addition, the patient’s headache persisted despite a trial of NSAIDs, acetaminophen, tramadol, and Fiorecet. Upon reconciliation of the patient's medications, it was found that she was switched from metformin 500 mg twice daily to metformin-sitagliptin 50-1000 almost a year ago, about a week before the onset of headache. Considering the temporal association of the medication and symptom presentation, sitagliptin was discontinued as a trial treatment and the patient was switched back to metformin. The patient reported resolution of her headache two days after discontinuation of sitagliptin.

## Discussion

Sitagliptin is the first DPP4 inhibitor to be approved for use in the United States. It is an incretin-based medication. Glucagon-like-peptide-1 (GLP-1), an incretin produced by GI L cells in response to nutrient absorption, stimulates insulin release by pancreatic β cells, inhibits glucagon secretion, and induces satiety resulting in weight loss or minimal gain. GLP-1 is quickly inactivated by the DPP4 enzyme. By selectively inhibiting this enzyme, sitagliptin increases the effect of GLP-1 in the post-prandial state.

Sitagliptin, as monotherapy, initial combination therapy, or add-on therapy, has been extensively studied in numerous clinical trials and is well tolerated with a mild toxicity profile [1]. The most common adverse effects include upper respiratory tract infection, nasopharyngitis, headache, and GI upset. Rare instances of acute pancreatitis have been reported in the literature, but whether sitagliptin is the causal agent remains to be substantiated [2]. In a meta-analysis of 19 studies, including 10,246 patients with type II diabetes who received either sitagliptin 100 mg/day or a comparator agent, there was no significant difference in the incidence of headaches in rate per 100 patient-years (5.8 vs. 5.6, 95% CI -0.7-1.4) [3]. In a 12-week dose evaluation study consisting of 555 patients, Hanefeld et al. noted an overall incidence of headache in 2.7% of patients receiving study drug, with the highest rates of headache occurring patients receiving the higher 50 mg and 100 mg dosing schedule (3.6% in both groups) [4]. Aschner et al. demonstrated the effects of sitagliptin at 100 mg or 200 mg dosing compared to placebo in 741 patients. They report headache in patients at 4.7%, 4.6%, and 4.4% in the placebo, sitagliptin 100 mg, and sitagliptin 200 mg arms of the study, respectively [5]. Importantly, none of the studies indicate the severity of the headaches experienced nor do they indicate that any patient was discontinued from sitagliptin therapy due to headache. Indeed, there were higher rates of reported headaches in the placebo arms of the study conducted by Aschner et al. This may indicate the mild nature of headaches reported in the treatment arms of this study. However, our case demonstrates the occurrence of unresponsive intractable chronic headache secondary to sitagliptin as evidenced by temporal association and symptom resolution on discontinuation of the medication.

## Conclusions

To the best of our knowledge, this is the first report of a patient discontinuing sitagliptin due to headache. Due to the widespread nature of type II diabetes in many areas of the world, anti-diabetic drugs with manageable side effect profiles are a clinical necessity. Given the acceptable safety profile of DPP4 inhibitors and their demonstrated efficacy, their clinical prevalence will likely continue to rise. Continued monitoring will be necessary to elucidate long-term side effects of the therapy and protect patients from unnecessary discomfort. Our case demonstrates the need for continuous monitoring and post-marketing surveillance even for drugs with a tolerable side effect profile. Also, increasing patient and physician awareness of long-term side effects of these commonly used medications is essential for adequate patient safety and quality care.
